# Evaluating City-Cut Geographic Risk Classification for a Pandemic Travel-Record System in Shanghai, China: Retrospective Observational Analysis

**DOI:** 10.2196/64941

**Published:** 2026-11-09

**Authors:** Rowan Wang, Baofeng Shi, Shenghan Li, Cong Xie, Qingpeng Zhang

**Affiliations:** 1 College of Business, Southern University of Science and Technology, Shenzhen, Guangdong, China; 2 College of Economics and Management, Northwest A&F University, Xianyang, Shaanxi, China; 3 Carlson School of Management, University of Minnesota Twin Cities, Minneapolis, MN, United States; 4 China Economic Research Institute, Liaoning University, Shenyang, Liaoning, China; 5 Department of Pharmacology and Pharmacy, LKS Faculty of Medicine, The University of Hong Kong, Pokfulam Road, Hong Kong, China (Hong Kong), 852 39102337; 6 Musketeers Foundation Institute of Data Science, The University of Hong Kong, Hong Kong, China (Hong Kong)

**Keywords:** COVID-19, human mobility, nonpharmaceutical intervention, epidemic modeling, epidemic control

## Abstract

**Background:**

Smartphone-delivered travel-record and health-code systems were deployed across China during the COVID-19 pandemic to classify risk based on travel history and regulate access to venues and travel. The geographic classification rule is a key design choice for these systems. A city-boundary rule (city-cut) is administratively convenient but may group areas with different mobility patterns under one risk label, whereas a fixed-radius rule (radius-cut) offers greater spatial precision but may exclude more distant areas connected by routine travel. Whether a classification rule aligns with observed mobility is a design question distinct from evaluating a deployed system’s epidemiological effects.

**Objective:**

This study examined whether observed mobility in Shanghai supported a city-cut or a radius-cut geographic design for a mobile health travel-record system and whether mobility-derived communities corresponded to administrative districts.

**Methods:**

We conducted a retrospective observational analysis of 1.44 billion deidentified China Unicom mobility records collected for two 46-day periods in 2019 and 2020. The analytical unit was the daily origin-area movement proportion. We calculated within-Shanghai, confirmed external-city, and nonadjacent-district movement proportions; for each origin area, we paired them by calendar day. We constructed grid-level transition matrices, used spectral clustering followed by k-means++, and checked the groupings across repeated initializations and selected dates.

**Results:**

Across 46 daily city-average observations in 2019, the intracity proportion was 97.2% (95% *t* CI 97.1%‐97.3%). Nonadjacent-district movement was 13.7% (95% *t* CI 13.5%‐13.9%), compared with 2.8% (95% *t* CI 2.7%‐2.9%) for confirmed external-city movement. A paired, 2-tailed Newey-West heteroskedasticity- and autocorrelation-consistent (HAC) *t* test (α=.05) gave a mean difference of 10.9 (95% HAC CI 10.7‐11.1; *P*<.001) percentage points. At the Chongming border, the corresponding difference was –3.1 (95% HAC CI –4.1 to –2.1; *P*<.001) percentage points.

**Conclusions:**

This study moves the question from postdeployment evaluation to predeployment design, treating geographic calibration as an upstream mobile health decision. It differs from prior work that assessed uptake, privacy, or epidemiological outcomes after fielding, and from mobility research that treated movement data as a predictor, an outcome, or a model input rather than as a criterion for choosing a boundary rule. The contribution is threefold: it formulates a boundary-fit question that can be answered before a system is built; it provides a reproducible, auditable workflow (daily origin-area proportions, a paired HAC-based comparison, and a mobility-clustering check) that agencies and developers can rerun on their own data; and it demonstrates the workflow from end to end in a large municipality. Practically, a city-level unit is a defensible starting reference where movement is predominantly internal, whereas border areas warrant separate local assessment. Agencies should document where a boundary fits local movement and where exceptions may be needed. Decisions about restrictions also require epidemiological evidence and assessment of proportionality, public acceptability, and social costs.

## Introduction

During the COVID-19 pandemic in mainland China, health-code and travel-record systems used smartphone-delivered information to regulate access to venues and travel [1-3]. These systems made the choice of geographic unit consequential: a city-level risk label could affect people whose recorded travel history included that city. Location-based digital health tools also raised concerns about privacy, consent, and governance [1,4-9]. A central design question is the spatial scale at which a travel-history system should assign a risk label. An administrative city boundary is simple to communicate and implement, whereas a fixed-radius rule is geographically narrower [1-3]. The best fit depends partly on how people move across and within the relevant boundaries. A broad label can encompass places with different movement patterns, while a narrow label can divide locations connected by routine travel. The design task is therefore to make the geographic assumption explicit and assess its consistency with observed connectivity before using it to classify travel histories.

We examine this question using mobility data from Shanghai, China. Shanghai is a large and internally heterogeneous municipality with 16 districts [10]. The city provides a useful comparison between a city boundary and a fixed-radius rule: residents may live far from an index location within Shanghai, while some locations just beyond the municipal boundary may be geographically close. This setting makes it necessary to distinguish geographic distance from membership in the same administrative unit. Examining the city as a whole, alongside areas near its municipal boundary, can reveal whether an aggregate pattern obscures local differences. The internal district structure also provides an administrative reference against which to consider functional mobility regions. We evaluate both designs against observed mobility without assuming that either applies universally.

First, we ask whether observed movement within Shanghai is more frequent than confirmed movement to other cities, including for border-origin areas. Second, we ask whether movement between nonadjacent Shanghai districts is substantial and more frequent than confirmed external-city movement, including for border-origin areas. We refer to these as the intracity and nonadjacent-district comparisons. The first concerns how much recorded movement remains within the proposed city unit. The second concerns connectivity across more widely separated parts of that unit, which is relevant to whether a geographically narrow label would capture the scale of routine movement. Border-origin areas are especially informative because proximity to another jurisdiction may weaken the fit of a uniform municipal label. Both comparisons describe mobility; neither tests infection transmission nor intervention effectiveness. These questions connect work on digital public-health systems with research on mobility and epidemic spread [11].

Location-based digital public-health tools, including digital contact tracing, geofenced quarantine monitoring, and travel-record systems, became prominent components of COVID-19 governance [12-14]. Mobile health (mHealth) scholarship identifies client registration, tracking of vital events, and data collection as core application types [15]. A scoping review distinguishes contact tracing and location-based risk assessment from population-level outbreak detection and mobility tracking [14]. This distinction helps locate the present question within digital public health: the geographic rule used to summarize a travel history should be evaluated in relation to that function, without treating it as a measure of individual contact or infection. Prior evaluations have examined adoption and epidemiological outcomes after deployment [7,16-19], while work on health-code systems has emphasized their operational and governance implications [1-3,6,20,21]. Studies in this literature show that design and governance shape how digital systems operate [6,8,9], but seldom test the geographic unit that determines who will be flagged before the system is deployed. Selecting the boundary before deployment differs from estimating whether a deployed tool changed infection outcomes. The question is whether a proposed spatial boundary fits observed mobility at the scale the system is intended to manage.

Large-scale mobile-phone datasets have been used to characterize human mobility during the COVID-19 pandemic, study the effects of movement restrictions, and inform public-health action [11,22-31]. Related research has examined spatial connectivity and the role of movement in infectious-disease models [32-36]. Work on the public-health use of mobile-phone data also emphasizes the need to match data and analysis to the decision being supported, while addressing access, coordination, and privacy constraints [26]. For a travel-record system, the relevant design question is whether the chosen unit captures observed connections within its boundary and distinguishes them from connections across it. This application requires an explicit link between a mobility measure and the geographic classification decision it is intended to inform. These studies generally treat mobility as a predictor, an outcome, or a model input rather than as evidence for choosing the geographic boundary of a digital travel-record system.

Network studies also show that interaction data can delineate functional regions that may differ from administrative borders [37,38]. Such functional regions summarize interaction patterns and provide a way to examine whether administrative divisions align with those patterns [37,38]. Alignment would support their use as an operational reference, whereas divergence would identify places where classification or coordination may require closer attention. Neither outcome alone establishes an appropriate restriction because a mobility partition and a public-health decision serve different purposes. Community-detection and spectral-clustering methods [39-42] can inform boundary design: mobility-based clustering can be compared with official geography, while aggregate movement shares can measure movement within and across a proposed unit. Mobility evidence cannot resolve legal, ethical, or epidemiological questions, but it can make the assumptions behind a geographic rule explicit.

To examine this design question, we use deidentified, aggregated Shanghai mobile-phone mobility records. We evaluate city-cut and radius-cut designs using daily intracity, confirmed external-city, border-origin, nonadjacent-district, and center-bound movement proportions. We also apply a prespecified spectral clustering procedure to assess how closely the resulting communities correspond to district boundaries. This approach evaluates geographic calibration before deployment and complements postdeployment evaluations of digital interventions. The study objectives are to assess whether within-city and nonadjacent-district mobility patterns in municipal-border areas support the use of a city-level geographic unit and to examine the correspondence between mobility-derived communities and administrative districts.

## Methods

### Study Context and Setting

This study evaluates the geographic design of China’s antipandemic travel-record card system in the Shanghai setting. The record card was a smartphone-based travel-history credential that displayed cities visited during the previous 14 days and could trigger restrictions when a listed city was classified as medium or high risk. Figure 1 illustrates the card interface used to communicate these city-level risk labels. Shanghai was selected because it is a large, highly connected municipality bordered by several major neighboring cities, making it a useful case for comparing city-cut and radius-cut risk classification. Figure 2 shows Shanghai’s district structure and surrounding cities, which define the geographic context for the mobility analysis.

**Figure 1.**
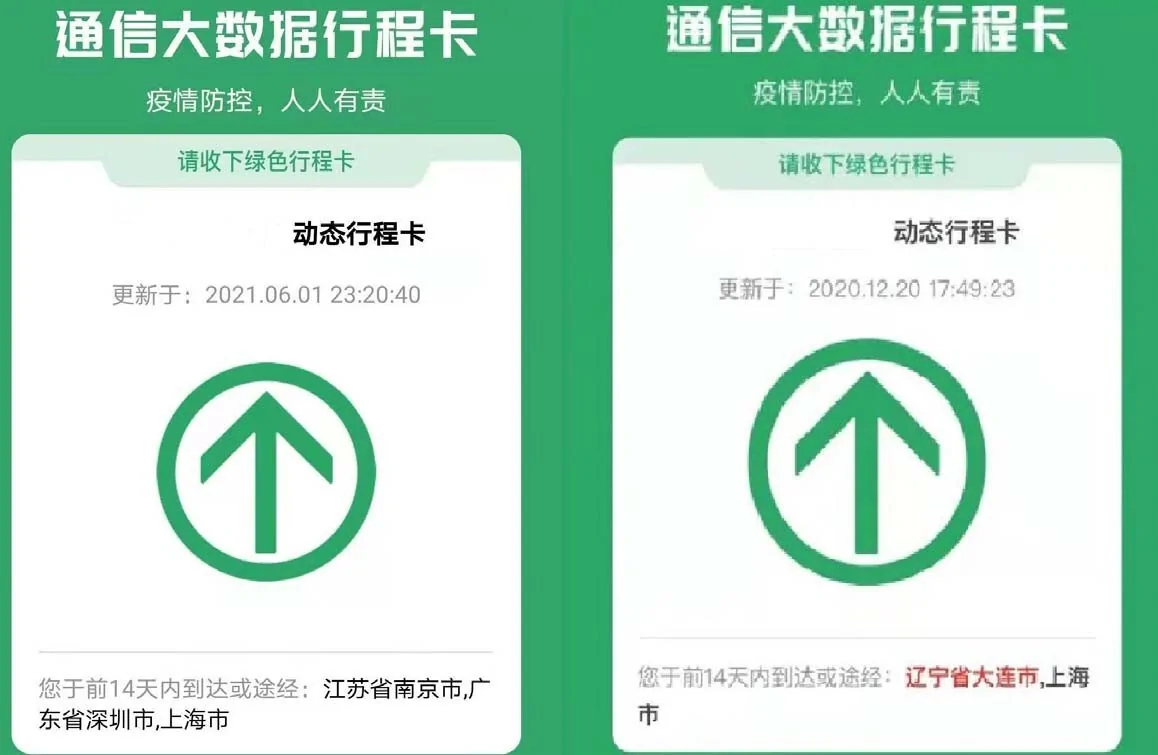
Chinese antipandemic travel-record card. Source: author-generated illustrative screenshot based on the record-card interface; no personal identifiers are displayed.

**Figure 2.**
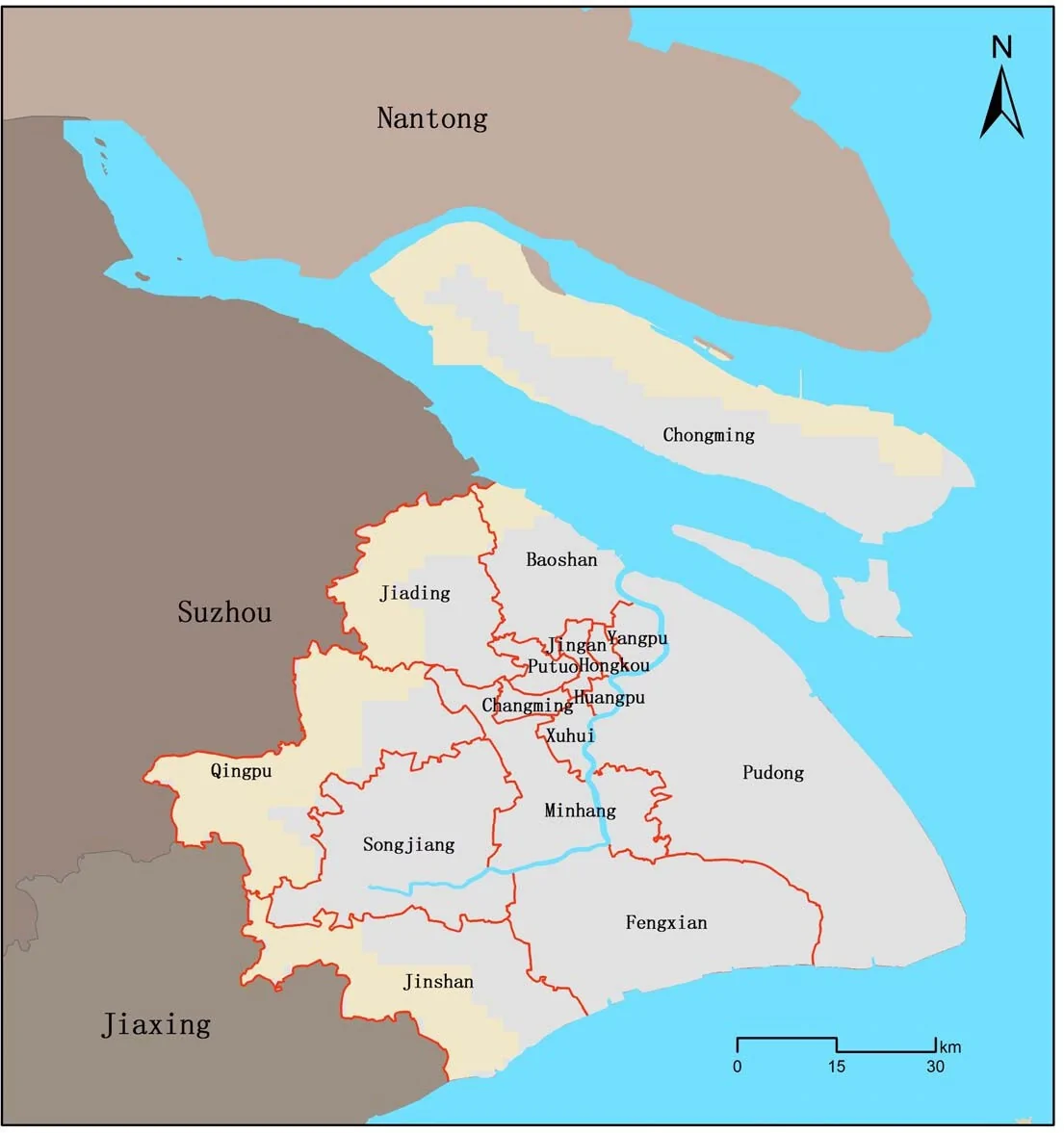
Map of Shanghai and surrounding areas. Source: author-generated map based on Shanghai administrative boundary data.

### Research Design

This was a retrospective observational secondary-data study. It involved no intervention allocation, randomization, or follow-up of individual participants. All conditions were naturally observed; the investigators did not manipulate mobility conditions or assign individuals or geographic areas to groups. The design comprised repeated daily observations during 2 prespecified 46-day windows in 2019 and 2020, as well as comparisons across naturally occurring geographic origin areas and movement categories.

### Data Collection Procedures

China Unicom processed and deidentified the supplied operator records before author access was granted. The authors received aggregated records for the prespecified 2019 and 2020 study windows and applied the documented visit, movement, and daily origin-area aggregation procedures.

### Inclusion and Exclusion

No participants were recruited. The provider preprocessed and deidentified the records before they were accessed by the authors. All supplied records within the study windows were used; the authors did not apply individual-level inclusion or exclusion criteria.

### Unit of Analysis and Data Characteristics

The unit of analysis was a daily origin-area movement proportion, not an individual participant. The study used deidentified, aggregated movement records and did not collect participant characteristics. The data did not contain individual names, phone numbers, device identifiers, or individual-level trajectories.

### Sampling Procedures

The dataset was supplied by China Unicom for the specified study windows in Shanghai. It is an operator-based data source rather than a probability sample of Shanghai residents. All obtained records were retained after provider deidentification and the documented movement-construction procedures.

### Sample Size, Power, and Precision

No a priori power analysis was performed because the analysis used all supplied aggregate records. Precision is reported using 2-tailed 95% *t* CIs for the mean of daily origin-area proportions across 46 days, based on day-to-day variation, with heteroskedasticity- and autocorrelation-consistent (HAC) intervals used as a serial-correlation-robustness analysis.

### Measures and Covariates

The prespecified mobility measures were intracity movement, confirmed external-city movement, nonadjacent-district movement, center-bound movement, and border-origin status. Administrative-district, center-area, and municipal-border labels were assigned through the grid crosswalk. No participant-level covariates were available or analyzed.

### Data Quality and Missing Data

No unavailable origin or destination codes were used in the analysis. State 9999 represented confirmed external-city journeys rather than missing geography, and no imputation was performed.

We obtained proprietary, deidentified, aggregated mobile-phone signaling records from China Unicom, a major Chinese mobile service provider. The data provide a large operator-based mobility dataset, but we do not assume that China Unicom subscribers represent the overall Shanghai population. This potential coverage bias is addressed as a study limitation.

China Unicom’s deidentified mobile-phone signaling records capture device connections to base stations, which were used to reconstruct aggregated daily visits and movement segments. The dataset covers all records supplied for Shanghai from October 10 to November 24, 2019, and the corresponding dates in 2020. The movement-location records used in the analysis did not include records with missing origin or destination codes. Confirmed movements outside Shanghai were instead represented by the external-city state (9999).

Based on suggestions from location-tracking experts at China Unicom, we used the following settings:

The map of Shanghai was divided into 2213 grid units of 2 km × 2 km. If a device remained continuously in a grid for more than 30 minutes, we counted this as a visit to that grid. This 30-minute threshold was used to exclude brief pass-through observations, such as travel by car or train, without a substantive stay. When a device had 2 consecutive visits (eg, grid A followed by grid B), we counted this as a movement from grid A to grid B. If a device left Shanghai and remained in another city for more than 30 minutes, we used its last observed grid in Shanghai (eg, grid A) and counted this as a movement from grid A to another city. This counting procedure was repeated for each calendar day.

If a device stayed in 1 grid (eg, grid A) throughout a calendar day, we counted this as local movement within grid A.

In total, the supplied dataset contained 1.44 billion observed movement segments. The main text reports the 2019 baseline results, and Multimedia Appendix 1 reports the parallel 2020 analysis.

Data preprocessing followed a clean-append-merge workflow implemented in Stata (StataCorp LLC). Outbound Shanghai journeys were aggregated by day and origin grid and coded with the confirmed external-city destination state (9999); inbound journeys were aggregated by day and destination grid and coded with the confirmed external-city origin state (9999). These records were appended to within-Shanghai grid-to-grid movements. The 30-minute active-population file was merged by day and origin grid, and movement probability was calculated as $prob\;=\;\frac{wnum}{pop30}$, where wnum is the movement count and pop30 is the corresponding 30-minute active population. Each movement record carried an origin and a destination grid identifier (*s_fnid_* and *e_fnid_*), where *fnid* is the numeric code of a 2-km grid cell. The reserved value 9999 denotes the external-city state rather than a Shanghai grid. For inbound records originating outside Shanghai (*s_fnid_*=9999), the external-state denominator was set using the archived population-scaling rule; outbound records leaving Shanghai (*e_fnid_*=9999) retained the 30-minute active-population denominator of their Shanghai origin grid. For reproducibility, the fixed external-city denominator used for inbound records (*s_fnid_*=9999) was constructed by scaling the total Shanghai grid active population by the ratio of the population outside Shanghai to the Shanghai population; the formula, parameter values, and reference date are provided in Multimedia Appendix 1. Finally, origin and destination grids were merged with the grid-to-administrative-region crosswalk and the center-area, border-band, and external-city labels.

Visit histories were processed for each calendar day. A stay of more than 30 minutes in a 2 km × 2 km grid was treated as a visit. Two consecutive visits generated 1 directed movement segment. A day with no grid change was counted as a local movement. A device observed to leave Shanghai and remain in another city for more than 30 minutes was recorded from its last Shanghai grid to a confirmed external-city state; this coding did not denote missing data.

### Statistical Analysis

We defined 4 movement types: intra was movement within Shanghai, including local movement; inter was movement to the confirmed external-city state; intra ld was movement between nonadjacent Shanghai districts; and intra tc was movement toward one of the 7 central districts (Changning, Hongkou, Huangpu, Jing’an, Putuo, Xuhui, and Yangpu).

The analytical unit was the daily origin-area proportion. For every district, border-origin area, and city-average series, we reconstructed 46 calendar-day proportions and reported their mean with a conventional 2-tailed 95% *t* CI, calculated from the same 46 daily proportions. As a robustness analysis for possible serial correlation across consecutive days, we also calculated Newey-West HAC intervals from an intercept-only model with lag 3, selected by the conventional rule $\left\lfloor 4{\left(\frac{N}{100}\right)}^{\frac{2}{9}}\right\rfloor$ for *N*=46 [43]. The HAC results were substantively consistent with the conventional 95% *t* CIs. Hypothesis comparisons used a paired, 2-tailed Newey-West HAC *t* test of the 46 daily differences, *d*_*t*_=Intra ld_*t*_-Inter_*t*_, with null mean (*d*_*t*_)=0 and α=.05. Movement segments were not treated as independent observations. Inference was based on the 46 ordered daily proportions; the HAC analysis allowed heteroskedasticity and autocorrelation through lag 3.

For a daily proportion series, the conventional interval was calculated as $\overset{-}{x}\;\pm \;t_{0.975,45}\frac{s}{\sqrt{46}}$, where $\overset{-}{x}$ is the mean of the 46 daily proportions and s is their SD. It is therefore a conventional descriptive interval based on day-to-day variation, not an individual-movement sampling interval. The Newey-West HAC covariance estimator adjusts the estimated uncertainty for heteroskedasticity and autocovariances through lag 3, reducing reliance on independence of adjacent daily proportions; it was reported as a robustness analysis rather than as proof that all temporal dependence had been removed [43,44].

City-border areas were defined as grids within Shanghai’s boundary districts that were within 8 km, equivalent to four 2-km grid units, of the municipal boundary. This definition is used consistently throughout the main analysis and in Multimedia Appendix 1. We then repeated the movement-proportion calculations for movements originating within these border areas.

The intracity long-distance metric is an operational measure of nonadjacent-district connectivity, not proof of a direct, nonstop trip. Because visits are recorded only after a stay of more than 30 minutes, a segment may include short, unrecorded pass-through travel, including through central areas. Conversely, a stay of more than 30 minutes in an intermediate grid splits a journey into separate segments.

### Cluster Analysis

We explored whether the tested mobility-clustering specification produced an administratively interpretable subcity partition. This was an exploratory analysis: it assessed the correspondence of a specified clustering procedure to district geography, rather than adjudicating among all possible subcity classifications.

We used a spectral Markov-chain clustering approach [41,42]. The primary input was the October 10, 2019, Shanghai grid-level transition dataset, with 2213 Shanghai grid states plus the confirmed external-city state (9999). We summed repeated origin-destination edges, constructed a sparse off-diagonal transition matrix from observed probabilities, and assigned the residual probability to each diagonal stay entry. States with no observed off-diagonal movement were removed, and the reduced row-stochastic matrix was rebuilt before eigendecomposition. We computed 8 eigenvectors using eigs(T2,8,‘largestabs’) and clustered the real-valued second eigenvector. The primary specification used *k*=16. In an audited reimplementation, k-means++ initialization, Lloyd iterations (maximum 1000), 100 restarts per seeded fit, and 50 fixed seeds (5000 initializations) were used. We also examined a 46-day transition-probability average and selected daily matrices across the 2019 and 2020 windows. Repeated fits were stable to initialization, but the tested specifications showed only partial and spatially heterogeneous correspondence with districts; they did not exactly reproduce the district partition and do not rule out other subcity classifications.

### Ethical Considerations

This study used secondary, deidentified, and aggregated mobile-phone signaling records provided by China Unicom under data-use and privacy restrictions. The authors did not recruit participants; interact with individuals; or access names, phone numbers, device identifiers, or individual-level trajectories. An institutional ethics review was not required for this secondary analysis because the dataset available to the authors had been deidentified before analysis, and all results are reported only as aggregated district-level, border-area, or grid-cluster summaries. No additional informed consent was obtained for this secondary analysis, and no compensation was provided because there was no participant recruitment or direct participation. The article, figures, and supplementary materials do not contain identifiable individual/user information.

## Results

### Unit of Analysis and Participant Flow

No participants were recruited or followed. The analytical flow comprised provider-supplied, deidentified movement records, daily origin-area aggregation, construction of 46 daily proportions for each series, and summary and paired comparison of those proportions.

### Recruitment

The study used secondary deidentified aggregate data and involved no recruitment, enrollment, or participant compensation.

### Statistics and Data Analysis

The exact inferential procedure, analytical unit, number of daily observations, pairing, sidedness, conventional 95% *t* CIs, and HAC robustness analysis are reported in the *Statistical Analysis* subsection. Tables 1 and 2 present the reconstructed means and conventional 95% *t* CIs.

**Table 1.** Movement proportions by Shanghai origin district, 2019.^a^

District	Intra^b^ (%), mean (95% CI)	Inter^c^ (%), mean (95% CI)	Intra ld^d^ (%), mean (95% CI)	Intra tc^e^ (%), mean (95% CI)
Baoshan	98.2 (98.2‐98.3)	1.8 (1.7‐1.8)	14.8 (14.5‐15.1)	15.2 (14.8‐15.7)
Changning	98.0 (97.8‐98.1)	2.0 (1.9‐2.2)	24.3 (24.1‐24.5)	18.6 (18.5‐18.8)
Chongming	93.7 (93.6‐93.8)	6.3 (6.2‐6.4)	11.2 (10.8‐11.5)	3.9 (3.7‐4.0)
Fengxian	99.0 (99.0‐99.1)	1.0 (0.9‐1.0)	4.7 (4.6‐4.8)	3.3 (3.2‐3.4)
Hongkou	98.5 (98.3‐98.7)	1.5 (1.3‐1.7)	15.1 (14.7‐15.4)	19.3 (18.9‐19.7)
Huangpu	98.5 (98.4‐98.6)	1.5 (1.4‐1.6)	23.4 (23.2‐23.6)	18.0 (17.8‐18.2)
Jiading	95.0 (94.9‐95.0)	5.0 (5.0‐5.1)	10.7 (10.6‐10.8)	11.4 (11.2‐11.7)
Jing’an	98.5 (98.4‐98.6)	1.5 (1.4‐1.6)	19.5 (19.3-19.7)	19.0 (18.7‐19.2)
Jinshan	94.7 (94.6‐94.8)	5.3 (5.2‐5.4)	9.9 (9.7‐10.1)	3.5 (3.3‐3.6)
Minhang	97.6 (97.5‐97.8)	2.4 (2.2‐2.5)	10.7 (10.4‐10.9)	13.1 (12.8‐13.4)
Pudong	98.1 (98.0‐98.1)	1.9 (1.9‐2.0)	10.1 (9.9‐10.2)	11.0 (10.7‐11.2)
Putuo	96.4 (96.1‐96.6)	3.6 (3.4‐3.9)	21.5 (21.1‐21.8)	18.6 (18.3‐19.0)
Qingpu	91.8 (91.4‐92.2)	8.2 (7.8‐8.6)	13.3 (13.1‐13.4)	8.2 (8.1‐8.3)
Songjiang	98.2 (98.1‐98.2)	1.8 (1.8‐1.9)	11.4 (11.2‐11.6)	7.1 (6.9‐7.2)
Xuhui	98.7 (98.6‐98.7)	1.3 (1.3‐1.4)	17.2 (17.0‐17.5)	19.0 (18.6‐19.4)
Yangpu	99.0 (98.9‐99.1)	1.0 (0.9‐1.1)	18.1 (17.8‐18.5)	18.8 (18.4‐19.1)
City average	97.2 (97.1‐97.3)	2.8 (2.7‐2.9)	13.7 (13.5‐13.9)	12.6 (12.4‐12.9)

a Values are means (conventional 95% *t* CIs) of 46 daily origin-area proportions. Corresponding Newey-West heteroskedasticity- and autocorrelation-consistent CIs, which allow for serial correlation across days, were evaluated as a robustness analysis.

b Intra: movements within Shanghai, including local movements.

c Inter: confirmed movements to the external-city state.

d Intra ld: movements between nonadjacent Shanghai districts.

e Intra tc: movements toward the 7 central districts.

**Table 2.** Movement proportions by Shanghai municipal-border origin area, 2019.^a^

Area	Intra^b^ (%), mean (95% CI)	Inter^c^ (%), mean (95% CI)	Intra ld^d^ (%), mean (95% CI)	Intra tc^e^ (%), mean (95% CI)
Baoshan city border	92.0 (91.9‐92.1)	8.0 (7.9‐8.1)	10.7 (10.6‐10.9)	5.3 (5.2‐5.5)
Chongming city border	84.6 (84.3‐84.9)	15.4 (15.1‐15.7)	12.3 (11.9‐12.7)	4.2 (4.0‐4.5)
Jiading city border	91.4 (91.3‐91.5)	8.6 (8.5‐8.7)	10.3 (10.2‐10.4)	8.9 (8.8‐9.0)
Jinshan city border	93.3 (93.2‐93.4)	6.7 (6.6‐6.8)	11.4 (11.2‐11.6)	4.1 (3.9‐4.2)
Qingpu city border	90.7 (90.5‐90.8)	9.3 (9.2‐9.5)	10.3 (10.1‐10.4)	5.8 (5.7‐5.8)
City average	91.0 (90.9‐91.1)	9.0 (8.9‐9.1)	10.8 (10.6‐11.0)	6.1 (6.0‐6.2)

a Values are means (conventional 95% *t* CIs) of 46 daily origin-area proportions. Corresponding Newey-West heteroskedasticity- and autocorrelation-consistent CIs, which allow for serial correlation across days, were evaluated as a robustness analysis.

b Intra: movements within Shanghai, including local movements.

c Inter: confirmed movements to the external-city state.

d Intra ld: movements between nonadjacent Shanghai districts.

e Intra tc: movements toward the 7 central districts.

### Ancillary Analyses

Ancillary analyses applied the same daily proportion workflow to a parallel 2020 window and examined the 46-day average and selected daily transition matrices. These analyses were used to assess the robustness of descriptive mobility and clustering patterns across dates.

### Adverse Events

This secondary analysis involved no interventions, participant recruitment, or participant-facing study procedures.

Table 1 reports mean daily mobility proportions for each origin district, and Table 2 reports the same quantities for movements originating in the 8-km municipal border band. Parentheses contain conventional 95% *t* CIs, based on 46 daily proportions rather than SDs. HAC intervals using the same daily series were examined as a serial-correlation-robustness analysis. The long-distance measure captures nonadjacent-district connectivity at the grid-day resolution and should not be interpreted as evidence that every movement was nonstop.

Across the 16 districts in 2019, intracity movement proportions exceeded 90%; the city average was 97.2% (conventional 95% *t* CI 97.1%‐97.3%). The city-average intracity long-distance proportion was 13.7% (conventional 95% *t* CI 13.5%‐13.9%), compared with 2.8% (conventional 95% *t* CI 2.7%‐2.9%) for confirmed external-city movement.

For district-origin series, the paired mean difference between intracity long-distance and external-city movement was positive in every district and at the city average. The city-average difference was 10.9 (95% HAC CI 10.7‐11.1; 2-tailed paired HAC *t* test *P*<.001) percentage points. The 2019 border-origin results were also positive except in Chongming, where external-city movement was more frequent than nonadjacent-district movement. The corresponding 2020 city-border average was not different from 0 (mean difference –0.1 percentage points, 95% HAC CI –0.3 to 0.1; *P*=.52), with negative differences in Chongming and Jiading. These exceptions qualify the citywide pattern and motivate local assessment near municipal boundaries.

The border-origin analysis is interpreted with deliberate caution. Chongming is geographically distinct, and its border-origin series had more confirmed external-city movement than nonadjacent-district movement in 2019. Therefore, border-area results do not support a uniform inference for every municipal edge.

Taken together, the movement results provide mobility-based evidence relevant to city-cut classification in Shanghai, while identifying boundary-area exceptions. They do not, by themselves, establish legal, ethical, social, epidemiological, or intervention-effectiveness legitimacy.

The same workflow was applied to the 2020 data; Multimedia Appendix 1 provides the 2020 point estimates and conventional 95% *t* CIs. The corresponding HAC analysis produced substantively consistent conclusions.

### Mobility Communities and District Geography

Figure 3 presents the 16-cluster spectral partition for the October 10, 2019, transition matrix. Colors identify mobility communities, and district boundaries are overlaid for comparison. The map shows partial geographic correspondence in several peripheral areas but no exact one-to-one recovery of all district boundaries. Repeated-initialization and selected-date checks produced stable partitions, but this tested procedure does not identify a unique optimal subcity partition. The corresponding 46-day average map is provided in Figure S1 in Multimedia Appendix 1.

The corresponding 46-day average partitions for 2019 and 2020 are presented in Figures S1 and S2 in Multimedia Appendix 1.

The clustering results are exploratory evidence of functional connectivity rather than a ready-made policy boundary. They are most informative when used to compare the tested mobility partition with existing administrative geography.

The single-day map shows broad, spatially coherent mobility regions, including partial correspondence with district geography in peripheral areas. In the 46-day average map (Figure S1 in Multimedia Appendix 1), several western areas remain visibly separated by district geography, whereas Fengxian and Pudong form a more integrated cross-district community, and the central urban area is more internally connected. These visual patterns identify places where cross-district coordination may be relevant; they do not confirm direct routes or infection transmission.

Some peripheral areas, including parts of Songjiang and Chongming, form more spatially concentrated blocks than the central city. Other blocks combine grids from multiple districts. This heterogeneity is consistent with districts being a practical first-order administrative reference in some settings, rather than a uniquely optimal or perfectly self-contained mobility partition.

The tested spectral-clustering specifications did not reproduce existing districts or simple district groupings as an exact map. This result does not rule out other subcity classifications, which would require purpose-built objectives, geographic constraints, and independent validation before policy use.

**Figure 3.**
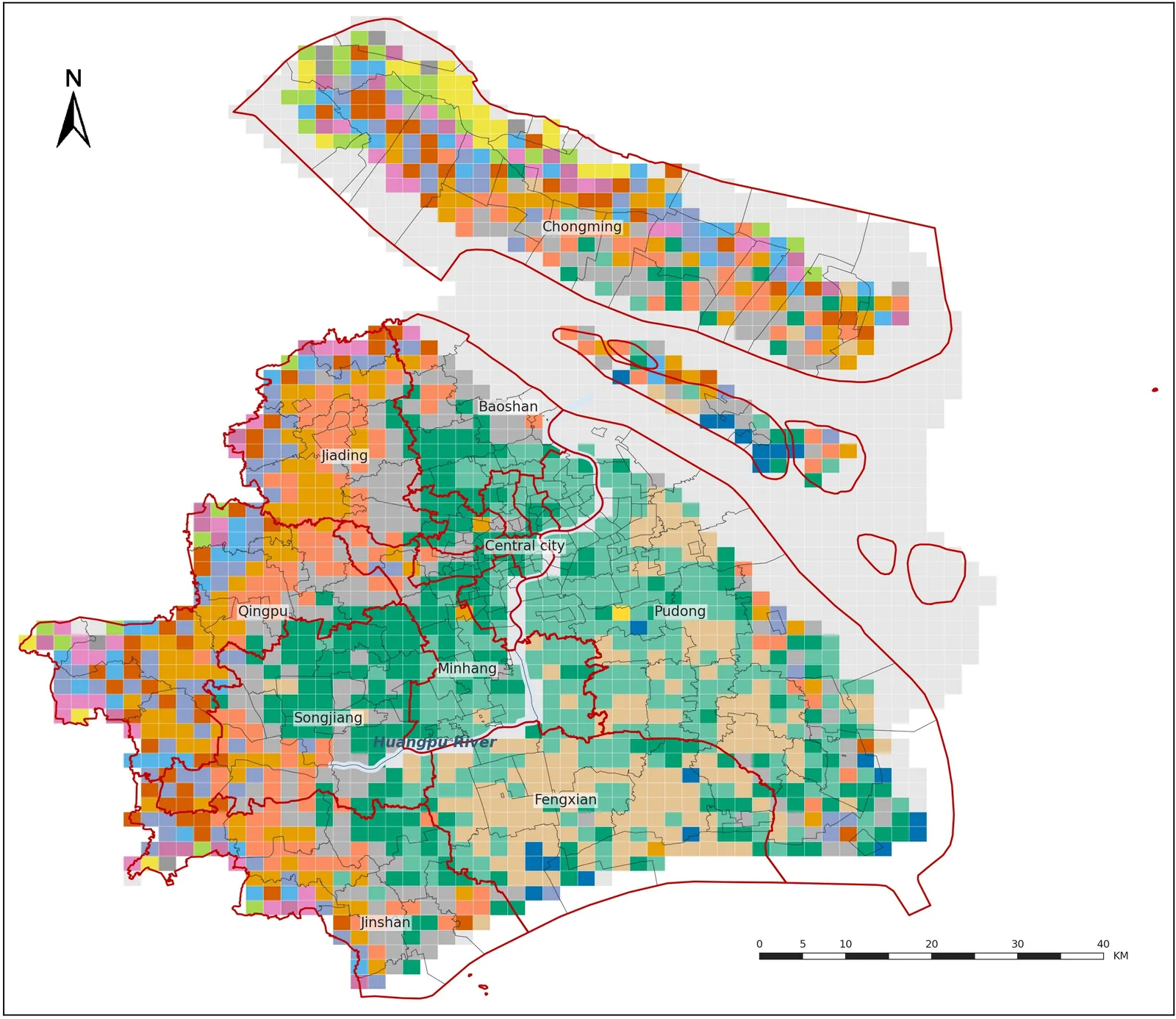
Spectral clustering of the October 10, 2019, Shanghai grid-level transition matrix (*k*=16). Colors identify mobility communities and district boundaries are overlaid. Source: authors’ calculations from deidentified China Unicom mobility records and Shanghai grid and district-boundary data.

## Discussion

### Principal Findings

This study examined whether daily mobility patterns provide evidence relevant to the city-cut geographic design of a digital travel-record system. In 2019, movement within Shanghai was common across all origin districts, and the city-average nonadjacent-district movement proportion exceeded the confirmed external-city movement by 10.9 (95% HAC CI 10.7‐11.1) percentage points. The 2020 analysis showed a similar district-level pattern, although border-origin comparisons were heterogeneous. These estimates concern observed mobility connectivity, not infection transmission or the effectiveness of a travel-restriction policy. The clustering analysis complements rather than overturns the city-level result. Under the reported spectral/Fiedler-vector/k-means specification, repeated fits produced stable mobility communities with partial and spatially heterogeneous correspondence to district geography. The 46-day average map retains some western district-oriented separation while showing tighter Fengxian-Pudong and central-area communities (Figure S1 in Multimedia Appendix 1). The clusters did not exactly reproduce the district map, but that finding neither rules out district-based management nor excludes other data-driven subcity classifications.

### Comparison With Prior Work

Prior mobility research has used digital-trace data to characterize movement, model spatial transmission, or evaluate how restrictions changed mobility [11,22-24,27-36]. Our study differs by using observed mobility to examine an upstream system-design choice: the geographic unit used to flag travel histories. This distinction is important because a boundary rule determines the spatial scope of a digital intervention before effects on transmission or uptake can be measured. Network and community-detection studies show that interaction data can reveal functional regions that sometimes diverge from official boundaries [37,38]. Building on that work, we use grid-level transition probabilities to compare a city boundary with a radius-based alternative and to assess whether a tested subcity clustering procedure corresponds to administratively usable districts. The contribution is an empirical, reproducible boundary-calibration workflow rather than a model of disease spread or a claim that mobility alone determines a policy’s value. The results should not be read as evidence that mobility connectivity causes rapid transmission, that city-cut policies reduce infections, or that city-wide restrictions are proportionate. Those questions require linked epidemiological, behavioral, legal, and equity evidence [6,16-19]. The present study instead documents a mobility pattern that policymakers could consider alongside those other forms of evidence when defining geographic risk units.

Location-based mHealth tools were widely deployed during the COVID-19 pandemic, and evaluations of these systems have examined uptake, privacy practices, and epidemiological outcomes [7-9,16-19]. Our analysis contributes earlier in the design sequence by making the geographic classification rule empirically inspectable. It shows how aggregate mobility data can be used to test whether a proposed administrative boundary is broadly consistent with the movement scale the tool is intended to manage. For Shanghai, high within-city mobility and substantial nonadjacent-district movement provide a mobility-based rationale for considering a city-level unit, with clear caution for some border-origin areas. At a subcity scale, districts offer an administratively available first-order reference, but central and corridor-like connections may need cross-district coordination. This interpretation is deliberately narrower than a claim of policy effectiveness or broad legitimacy.

### Real-World Implications

The most direct real-world application of this workflow is at the point where a location-based mHealth system is specified rather than after it has been fielded. Travel-record and health-code systems require a geographic unit before a single record can be classified, and that unit determines who will be flagged [1-3,20,21]. The analysis reported here shows that this choice can be treated as an explicit, testable parameter: daily origin-area movement proportions and a paired HAC comparison give a documented and reproducible basis for adopting or rejecting a candidate boundary. Development teams can therefore record the mobility assumptions behind a geographic rule in the same way they record data retention or consent settings, producing an auditable design trail that reviewers, regulators, and the public can inspect [8,9]. For public-health agencies, the results support treating a city-level boundary as a first-order operational reference in settings where observed movement is predominantly internal. In Shanghai in 2019, the city-average intracity proportion was 97.2% (95% *t* CI 97.1%‐97.3%) and nonadjacent-district movement exceeded confirmed external-city movement by 10.9 (95% HAC CI 10.7‐11.1) percentage points, so the movement that a city-level rule is intended to govern occurred largely inside the boundary that the rule would draw. Agencies elsewhere can apply the same daily proportion construction to test whether that condition holds locally before adopting a comparable rule and can repeat the test as travel patterns change, rather than importing a boundary convention from another jurisdiction.

The border-origin results carry a more specific operational message. Comparisons for the 8-km municipal border band were heterogeneous, and Chongming showed a negative difference, meaning that confirmed external-city movement exceeded nonadjacent-district movement for that origin area. A uniform city label would misdescribe the movement of such areas. Practical responses include treating designated border zones as separate classification units, applying a lower-intensity label within them, or coordinating classification with the adjacent jurisdiction. The clustering results point the same way at a finer scale: mobility communities showed only partial correspondence with district geography, so central and corridor-like areas may warrant cross-district coordination even where a city-level unit is retained [37,38].

More importantly, the workflow is not specific to Shanghai, to a single operator’s data, or to COVID-19. It requires only aggregated origin-destination counts, an activity denominator, and a candidate boundary, inputs that are available to operators and statistical agencies in many settings [25,26]. It can be applied to other administrative units, to other hazards for which movement-based risk labeling is proposed, and to periodic recalibration as travel patterns shift. Where comparable data cannot be obtained, the same reasoning can still be used qualitatively to state, and later test, the mobility assumption on which a proposed boundary depends. At last, these implications should be read within the study’s evidentiary limits. The analysis describes observed mobility. It does not measure infections, evaluate whether a city-cut rule reduced transmission, or establish that any restriction was legally authorized, ethically proportionate, or publicly acceptable [16-19]. Mobility evidence is one input among several, and this workflow is offered as a way to make that one input explicit and checkable, not as a decision procedure for policy.

### Strengths and Limitations

This study’s strengths are the large deidentified operator dataset, the reconstruction of daily origin-area proportions, the parallel 2019 and 2020 analyses, and transparent operational definitions for the movement measures. The grid-level records allow movement to be examined across Shanghai while retaining the spatial detail needed to distinguish district and municipal-border patterns. Applying the same geographic definitions and daily aggregation procedures in both years provides a consistent basis for comparing the 2 study periods. The district-level and border-origin comparisons help reveal local differences that would be obscured by a citywide average, while the clustering analysis provides a complementary view of how observed connectivity relates to administrative geography. Documenting the visit threshold, movement categories, and transition-matrix construction makes the analytical choices explicit and supports the assessment of the approach in settings with comparable data.

Despite these strengths, several limitations should be acknowledged. First, reliance on 1 mobile service provider may introduce bias if its users differ from the broader population, despite China Unicom’s substantial market share [35]. Second, the visit definition requires a stay of more than 30 minutes in a 2 km × 2 km grid. This threshold reduces pass-through noise but may miss shorter epidemiologically relevant encounters. Third, the nonadjacent-district metric captures connectivity at the resolution of recorded visits and may include unobserved pass-through travel. Fourth, this study provides a mobility-based empirical justification for city-cut classification, but it does not evaluate epidemiological effectiveness, legal authority, ethical proportionality, public acceptability, or the social costs of restrictions. Future research should combine mobility data with epidemiological and policy-outcome data to assess these broader dimensions.

### Conclusions

This study introduces a mobility-based approach to an upstream design choice in location-based mHealth: how to define the geographic unit used for travel-risk classification. Unlike studies that assess transmission or intervention outcomes after deployment, it uses daily grid-to-grid mobility to assess whether a proposed boundary is consistent with observed movement. In Shanghai, the results support considering a city-level unit as a mobility-based reference while identifying border-area and subcity exceptions that require local assessment. The approach can inform more transparent geographic calibration of digital public-health tools, but it does not establish infection effects, legal legitimacy, or the overall appropriateness of any restriction policy. The innovation of this work lies in relocating the analytic question from postdeployment evaluation to predeployment design. It differs from existing studies in 2 respects. Evaluations of deployed location-based mHealth tools have assessed uptake, privacy practices, and epidemiological outcomes [7-9,16-19], and prior mobility research has treated movement data as a predictor, an outcome, or a model input rather than as a criterion for choosing a boundary rule [11,22-24,27-36]. This study contributes to the field a reproducible and auditable calibration workflow, combining daily origin-area movement proportions with paired HAC inference and transition-matrix clustering that can be applied to any candidate geographic unit in any setting with comparable aggregated mobility data. In real-world terms, the findings indicate that where observed movement is predominantly internal, a city-level unit is a defensible starting reference for travel-risk classification, while border-origin areas such as Chongming require separate local assessment before a uniform city label is applied, and areas whose mobility communities span administrative lines may require cross-district coordination. For developers and public-health agencies, the practical implication is that a boundary rule can be stated, tested, and documented before deployment rather than justified retrospectively.

## Supplementary material

10.2196/64941 Multimedia Appendix 1 This appendix reports the parallel 2020 analysis using the same 46-day daily proportion construction and conventional 95% *t* CIs. Corresponding heteroskedasticity- and autocorrelation-consistent analyses are reported as robustness checks.

## Abbreviations

HAC: heteroskedasticity- and autocorrelation-consistent
mHealth: mobile health
