# Supplementary material for: Evaluating City-Cut Geographic Risk Classification for a Pandemic Travel-Record System in Shanghai, China: Retrospective Observational Analysis
Source: JMIR Mhealth Uhealth. 2026 Nov 9;14:e64941. doi: 10.2196/64941 (PMC13747316; doi:10.2196/64941)
Supplement: Supplementary file 1 [file mhealth-v14-e64941-s001.docx]

# Multimedia Appendix 1

This appendix reports the parallel 2020 analysis using the same 46-day daily-proportion construction and conventional 95% t confidence intervals; corresponding HAC analyses are reported as robustness checks.

**Table S1. Movement proportions by Shanghai origin district, 2020**

| District | Intra | Inter | Intra ld | Intra tc |
| --- | --- | --- | --- | --- |
| Baoshan | 98.3% (98.3%-98.4%) | 1.7% (1.6%-1.7%) | 14.1% (13.8%-14.4%) | 14.9% (14.4%-15.4%) |
| Changning | 97.9% (97.8%-98.0%) | 2.1% (2.0%-2.2%) | 23.5% (23.2%-23.8%) | 17.7% (17.5%-17.9%) |
| Chongming | 93.3% (93.2%-93.4%) | 6.7% (6.6%-6.8%) | 9.6% (9.2%-9.9%) | 2.7% (2.6%-2.8%) |
| Fengxian | 99.1% (99.0%-99.1%) | 0.9% (0.9%-1.0%) | 4.7% (4.6%-4.7%) | 3.0% (2.9%-3.0%) |
| Hongkou | 99.2% (99.2%-99.3%) | 0.8% (0.7%-0.8%) | 15.1% (14.6%-15.5%) | 19.2% (18.7%-19.7%) |
| Huangpu | 98.9% (98.8%-98.9%) | 1.1% (1.1%-1.2%) | 23.1% (22.9%-23.4%) | 17.6% (17.3%-17.9%) |
| Jiading | 94.3% (94.3%-94.4%) | 5.7% (5.6%-5.7%) | 10.3% (10.2%-10.5%) | 10.6% (10.4%-10.9%) |
| Jingan | 98.8% (98.8%-98.9%) | 1.2% (1.1%-1.2%) | 19.7% (19.4%-20.0%) | 18.4% (18.1%-18.7%) |
| Jinshan | 94.0% (94.0%-94.1%) | 6.0% (5.9%-6.0%) | 8.7% (8.5%-8.8%) | 2.7% (2.6%-2.7%) |
| Minhang | 96.9% (96.8%-97.0%) | 3.1% (3.0%-3.2%) | 9.8% (9.6%-10.1%) | 12.5% (12.2%-12.8%) |
| Pudong | 98.3% (98.2%-98.3%) | 1.7% (1.7%-1.8%) | 9.6% (9.5%-9.8%) | 10.7% (10.3%-11.0%) |
| Putuo | 97.4% (97.3%-97.5%) | 2.6% (2.5%-2.7%) | 21.4% (20.9%-21.9%) | 17.7% (17.3%-18.1%) |
| Qingpu | 92.5% (92.4%-92.6%) | 7.5% (7.4%-7.6%) | 13.0% (12.9%-13.2%) | 8.0% (7.9%-8.2%) |
| Songjiang | 98.1% (98.1%-98.2%) | 1.9% (1.8%-1.9%) | 11.3% (11.1%-11.4%) | 6.9% (6.8%-7.1%) |
| Xuhui | 98.6% (98.5%-98.6%) | 1.4% (1.4%-1.5%) | 15.6% (15.3%-15.9%) | 16.5% (16.1%-16.9%) |
| Yangpu | 99.3% (99.2%-99.3%) | 0.7% (0.7%-0.8%) | 18.3% (17.8%-18.8%) | 18.7% (18.2%-19.2%) |
| City Average | 97.2% (97.2%-97.3%) | 2.8% (2.7%-2.8%) | 13.1% (12.9%-13.3%) | 12.0% (11.7%-12.3%) |

Note: Intra = movements within Shanghai, including local movements; Inter = confirmed movements to the external-city state; Intra ld = movements between nonadjacent Shanghai districts; Intra tc = movements toward the seven central districts. Values are means (conventional 95% t CIs) of 46 daily origin-area proportions. Corresponding Newey-West HAC CIs, which allow serial correlation across days, were evaluated as a robustness analysis.

**Table S2. Movement proportions by Shanghai municipal-border origin area, 2020**

| Area | Intra | Inter | Intra ld | Intra tc |
| --- | --- | --- | --- | --- |
| Baoshan city border | 91.6% (91.4%-91.7%) | 8.4% (8.3%-8.6%) | 8.6% (8.5%-8.7%) | 4.7% (4.6%-4.8%) |
| Chongming city border | 82.2% (82.1%-82.4%) | 17.8% (17.6%-17.9%) | 10.3% (10.0%-10.6%) | 2.8% (2.7%-2.9%) |
| Jiading city border | 89.6% (89.5%-89.7%) | 10.4% (10.3%-10.5%) | 9.9% (9.7%-10.1%) | 8.1% (7.9%-8.2%) |
| Jinshan city border | 92.3% (92.3%-92.4%) | 7.7% (7.6%-7.7%) | 10.0% (9.8%-10.2%) | 3.2% (3.1%-3.3%) |
| Qingpu city border | 90.4% (90.3%-90.5%) | 9.6% (9.5%-9.7%) | 9.9% (9.7%-10.0%) | 5.5% (5.4%-5.5%) |
| City Average | 90.0% (89.9%-90.1%) | 10.0% (9.9%-10.1%) | 9.9% (9.7%-10.1%) | 5.5% (5.4%-5.6%) |

Note: Intra = movements within Shanghai, including local movements; Inter = confirmed movements to the external-city state; Intra ld = movements between nonadjacent Shanghai districts; Intra tc = movements toward the seven central districts. Values are means (conventional 95% t CIs) of 46 daily origin-area proportions. Corresponding Newey-West HAC CIs, which allow serial correlation across days, were evaluated as a robustness analysis.

## Data Processing and Reproducibility

### Data Preparation and Spatial Definition

Raw China Unicom mobility files were processed in Stata. Shanghai was represented as 2-km grids. The center area comprised Changning, Hongkou, Huangpu, Jing'an, Putuo, Xuhui, and Yangpu. The municipal border band comprised grids in boundary districts located within 8 km, or four grid units, of the municipal boundary. The same definitions were applied in the main text and the 2020 supplement.

### External-City State Denominator

The external-city state (s_fnid = 9999) represented confirmed inbound journeys from outside Shanghai and did not correspond to a Shanghai grid. It therefore had no grid-specific 30-minute active-population denominator. Following the archived preprocessing implementation, this state was assigned a fixed denominator: pop30_external = [(1,400,000,000 - 24,281,400) / 24,281,400] x 35,732,160. The value of 1,400,000,000 denotes the national population total used in the archived code, and 24,281,400 denotes the Shanghai population. Thus, (1,400,000,000 - 24,281,400) / 24,281,400 is the ratio of the population outside Shanghai to the Shanghai population. The value of 35,732,160 was the Shanghai grid active-population reference total used in the archived implementation for October 10, 2019. This fixed denominator was applied to all inbound external-city records across study dates, and their movement probabilities were calculated as prob = wnum / pop30_external. It was a population-scaling construction for the external-city state rather than an observed daily active-population count outside Shanghai. Outbound records (e_fnid = 9999) retained the 30-minute active-population denominator of their Shanghai origin grid. An auxiliary sensitivity analysis varied the national-population input in the external-city denominator from 1.30 to 1.50 billion, changing the denominator by 7.3% in either direction. The Shanghai-grid second eigenvector remained near-perfectly correlated with the baseline (absolute correlation at least 0.99999999), and the 16-group partition had an adjusted Rand index of at least 0.983 relative to the baseline; at most 17 of 1,857 active Shanghai states changed matched cluster membership.

### Markov Transition Framework

For each day, directed movement records were aggregated by origin and destination state. Repeated state edges were summed, off-diagonal probabilities were constructed from the active-population denominator, and residual diagonal probability represented the stay component. The external-city state represented confirmed journeys outside Shanghai rather than unavailable region codes.

The transition probabilities were modeled in MATLAB as a sparse Markov transition matrix *T*. Each row represented an origin grid or the external-city state, and each column represented a destination grid or the external-city state. Observed transition probabilities from the Stata-prepared grid-level dataset were inserted into T, and diagonal entries were set to $T_{i,i} = 1 - \sum_{j} T_{i,j} (j \neq i)$to capture the remaining stay probability. States with diagonal value equal to 1, indicating no observed outgoing movement in the transition dataset, were removed before spectral decomposition.

### Community Detection via Spectral Clustering

The clustering procedure used the real-valued second eigenvector of the reconstructed transition matrix as the one-dimensional k-means feature. The audited implementation and its initialization, replicate, and selected-date checks are described in the main Methods section.

Figure S1. Spectral clustering of the 46-day average 2019 Shanghai grid-level transition-probability matrix ($k = 16$). Colors identify mobility communities and district boundaries are overlaid. Source: authors' calculations from deidentified China Unicom mobility records and Shanghai grid and district-boundary data.

Figure S2. Spectral clustering of the 46-day average 2020 Shanghai grid-level transition-probability matrix ($k = 16$). Colors identify mobility communities and district boundaries are overlaid. Source: authors' calculations from deidentified China Unicom mobility records and Shanghai grid and district-boundary data.

## Data Processing and Variable Construction

This appendix documents the Stata workflow used to process the 2019-2020 Shanghai 2-km-grid mobility data and construct the movement measures.

### Data Integration and Preprocessing

The primary data comprise grid-level inflow, outflow, and within-Shanghai movement records. The workflow aggregated raw records at the day-grid level, appended inbound and outbound records to within-city movements, merged the 30-minute active-population denominator, applied the documented external-flow scaling rule, and merged the grid-to-district and functional-zone crosswalk.

### Identification of Nonadjacent-District Movement

A movement was coded as nonadjacent-district movement when its Shanghai origin and destination districts did not share a boundary. This definition avoids classifying short cross-border commuting between adjacent districts as long-distance movement. It is an operational connectivity measure at the visit-recording resolution, not a direct-route measure.

### Mobility Probability Construction

For each origin-day record, mobility probability was calculated as movement volume divided by the corresponding 30-minute active population. District, border-band, and city-average statistics were then calculated from the appropriate origin-area denominator before daily aggregation.
